# Supplementary material for: A20 critically controls microglia activation and inhibits inflammasome-dependent neuroinflammation
Source: Nat Commun. 2018 May 23;9:2036. doi: 10.1038/s41467-018-04376-5 (PMC5964249; doi:10.1038/s41467-018-04376-5)
Supplement: Supplementary file 1 — Supplementary Information [file 41467_2018_4376_MOESM1_ESM.pdf]

Supplementary Information

A20 critically controls microglia activation and inhibits inflammasome-dependent neuroinflammation

Voet *et. al.*

Supplementary Figure 1

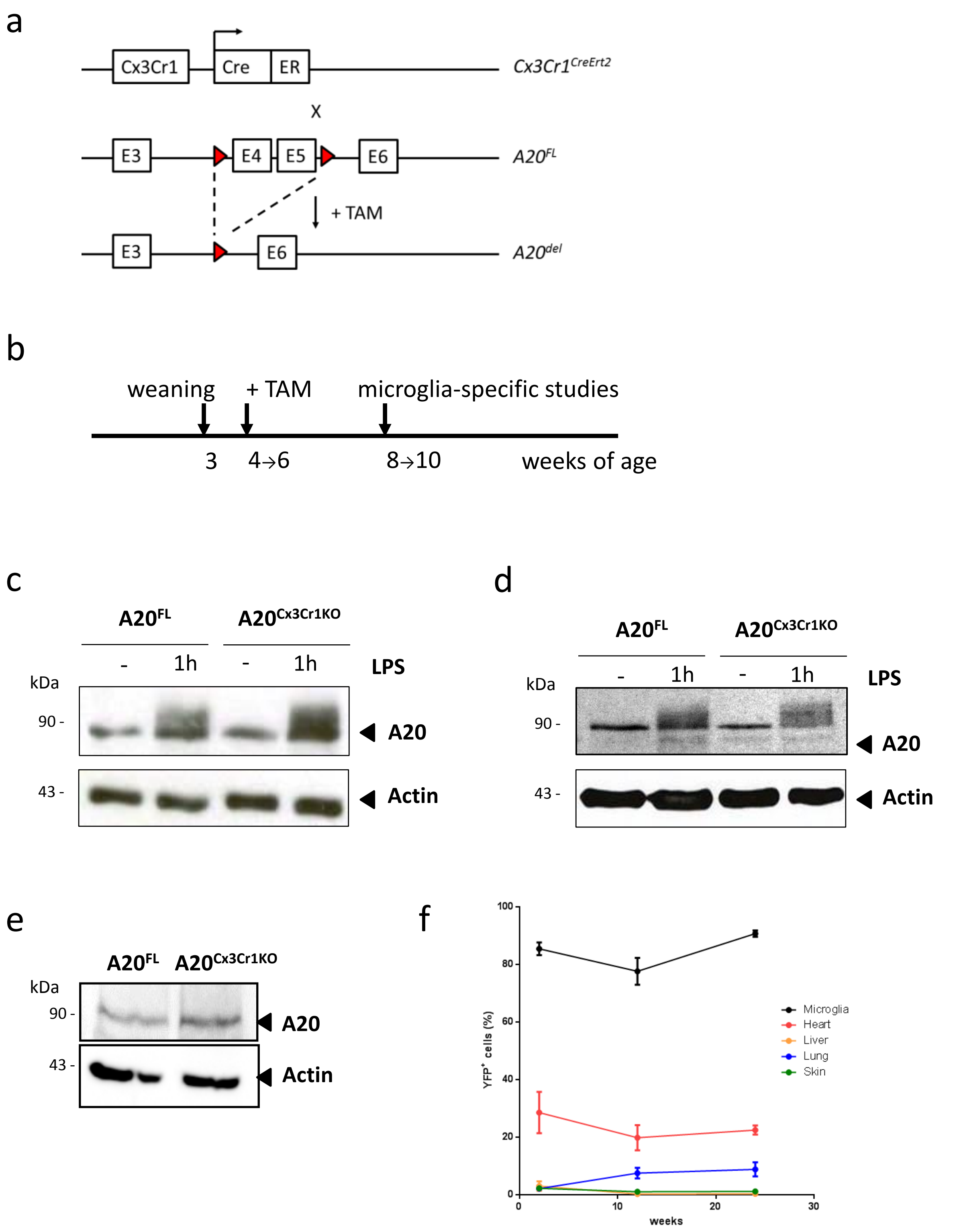

Supplementary figure 1. (a) Scheme for the induction of recombination and A20 deletion in inducible Cx3Cr1CreER:A20^{FL} animals. **(b)** TAM was given subcutaneously to 4 to 6-week-old animals and experiments were performed 4 weeks later. **(c)** Immunoblot depicting A20 expression in primary cultured BMDMs from control (A20^{FL}) and A20$^{Cx3Cr1\text{-}KO}$ mice 4 weeks after TAM injection, unstimulated or stimulated with LPS *in vitro*. Actin is shown as loading control. Data are representative of two independent experiments. **(d)** Immunoblot for A20 expression in primary cultured peritoneal macrophages from control (A20^{FL}) and A20$^{Cx3Cr1\text{-}KO}$ mice 4 weeks after TAM injection, unstimulated or stimulated with LPS *in vitro*. Actin is shown as loading control. Data are representative of two independent experiments. **(e)** Immunoblot for A20 expression in FACS-isolated Kupffer cells from control (A20^{FL}) and A20$^{Cx3Cr1\text{-}KO}$ mice 4 weeks after TAM injection. Actin is shown as loading control. **(f)** Flow cytometric quantifications of YFP^{+} CD64^{+}CD11b^{+} macrophages in the brain, liver, lung, heart and skin at the indicated time points after TAM application in Cx3cr1Ert2Cre:R26-YFP reporter mice. Data are presented as mean ± SEM.

Supplementary Figure 2

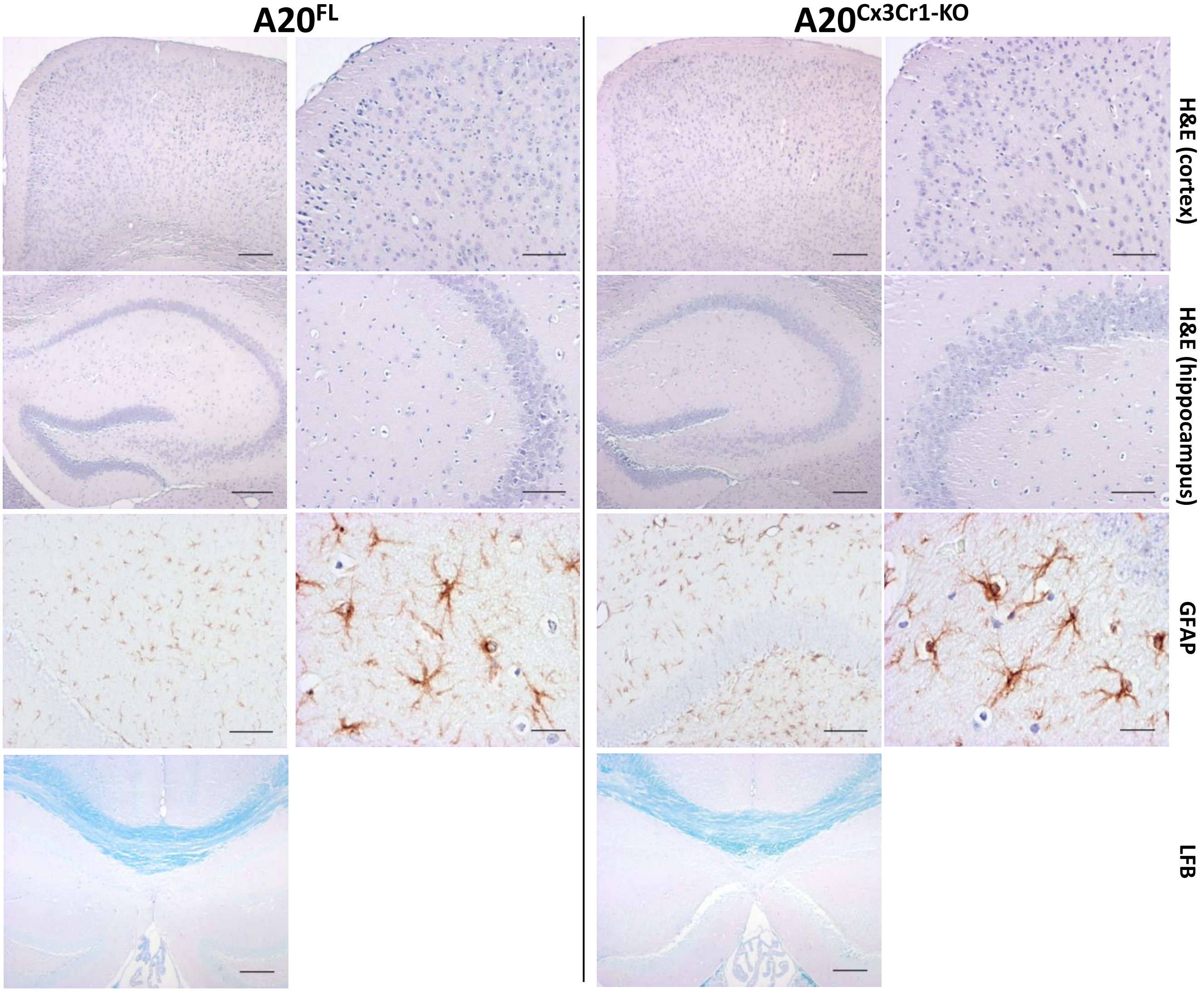

Supplementary figure 2. Absence of any gross abnormalities within the brain of A20$^{Cx3Cr1\text{-}KO}$ mice. H&E staining did not reveal any gross morphological defects in the cortex and hippocampus of 9-12 week old A20$^{Cx3Cr1\text{-}KO}$ mice (4 weeks after TAM injection). Scale bars represent 200 µm (overview) and 100 µm (zoom). GFAP immunohistochemistry revealed no signs of astrogliosis (scale bars represent 100 µm (overview) and 20 µm (zoom)). Luxol Fast Blue (LFB) staining revealed no signs of demyelination (scale bars represent 200 µm). Representative images are displayed.

Supplementary Figure 3

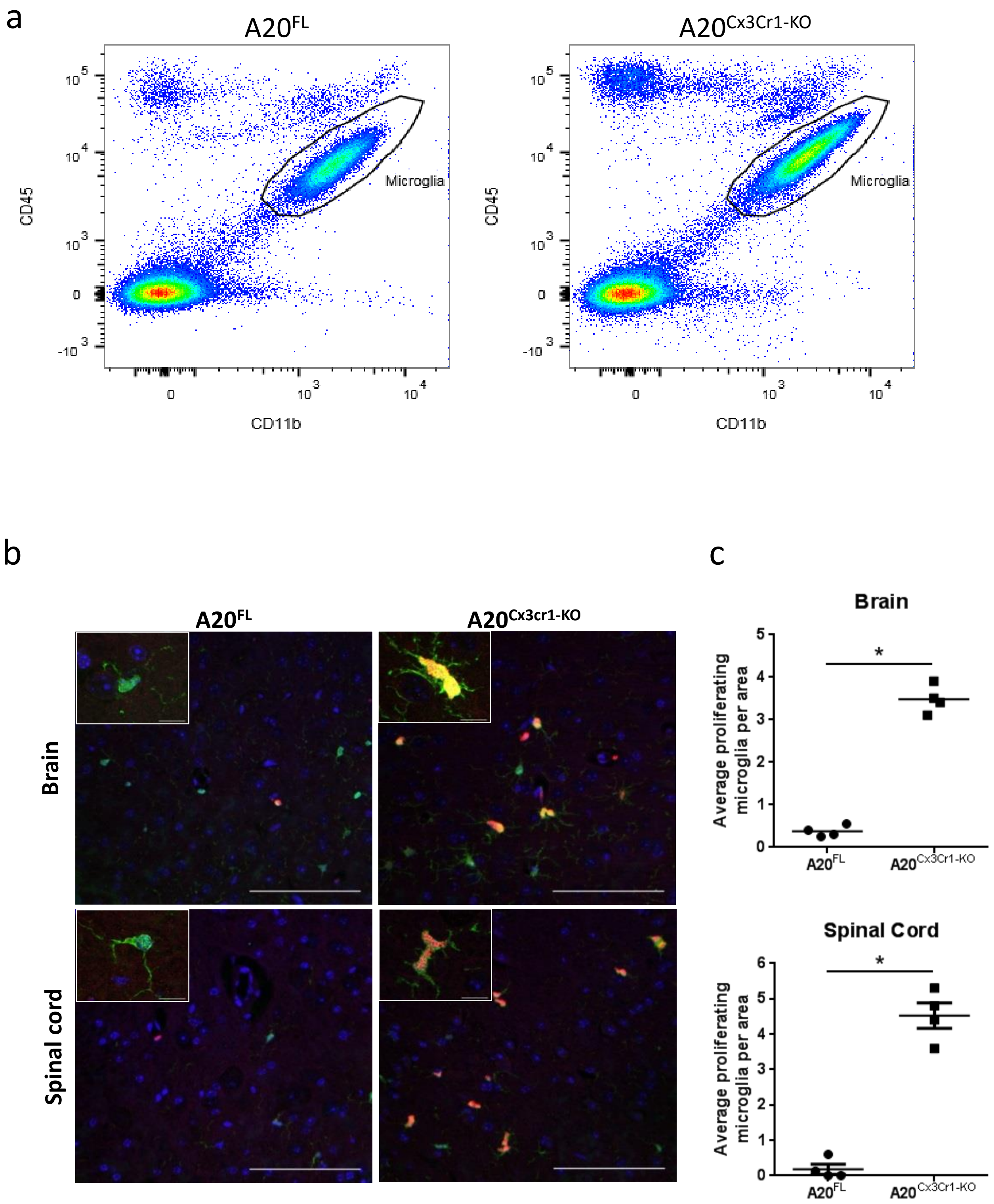

Supplementary figure 3. (a) Flow cytometric gating of CD11b$^+$ CD45^{int} microglia in the brain of control (A20^{FL}) and A20$^{Cx3Cr1\text{-}KO}$ mice 4 weeks after TAM injection. Representative dot plot for A20^{FL} and A20$^{Cx3Cr1\text{-}KO}$ mice. Data are representative of two independent experiments. **(b)** Immunofluorescent analysis of microglial cell proliferation 1 week post TAM injection showing Iba-1$^+$ (green) microglia, Ki-67 (red) as proliferation marker, and Hoechst (blue) in the brain and spinal cord of control (A20^{FL}) and A20$^{Cx3Cr1\text{-}KO}$ mice. Scale bars represent 100 µm (overview) and 20 µm (zoom). **(c)** Quantification of proliferating Iba-1$^+$ Ki67$^+$ double-positive parenchymal microglia in brain and spinal cord. Each symbol represents one mouse; n=4 per group. Seven to twelve images per mouse were examined. Data are represented as mean ± SEM. Significant differences were determined by a Mann-Whitney U statistical test (* $p < 0.05$).

Supplementary Figure 4

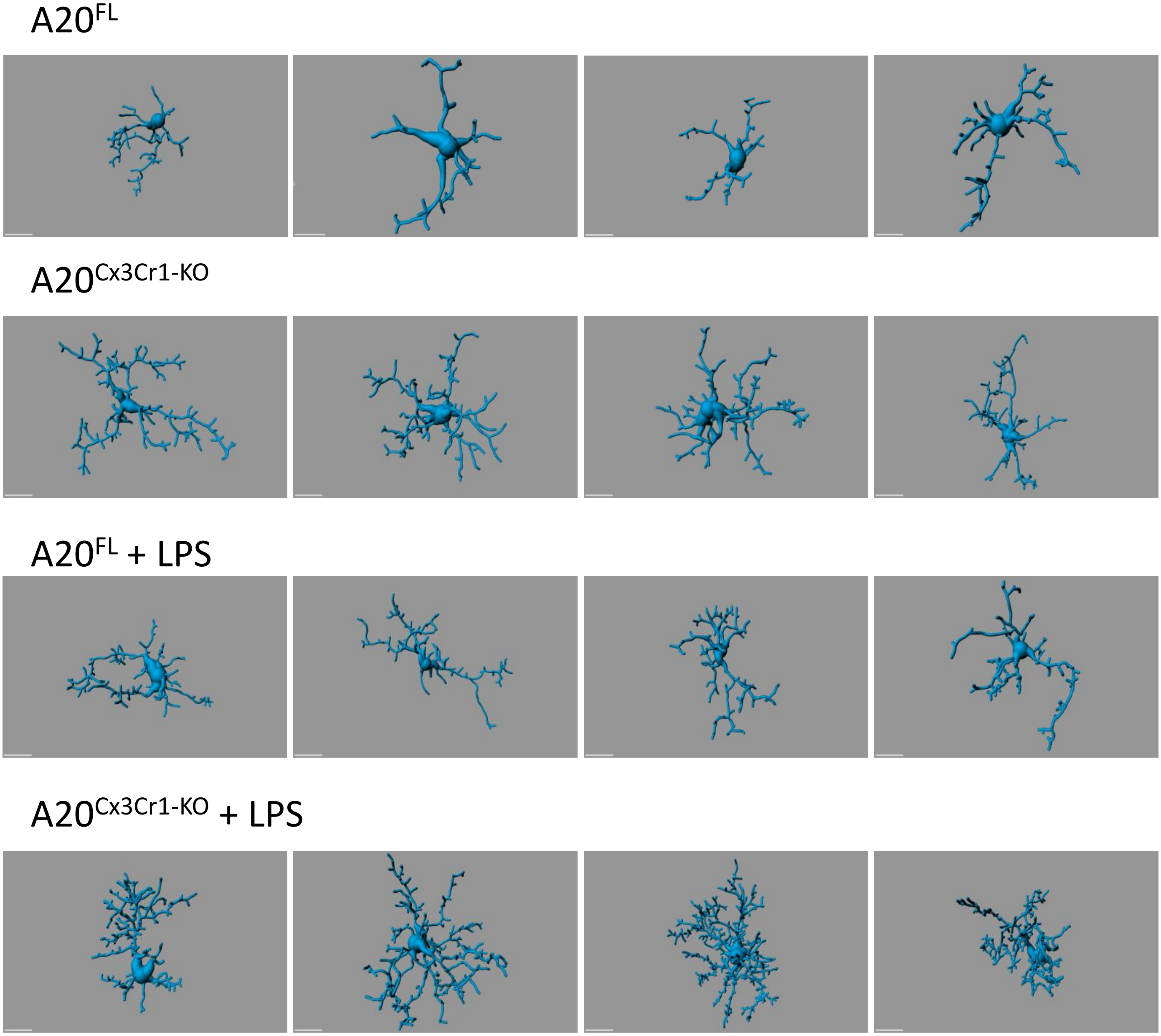

Supplementary figure 4. Three-dimensional reconstruction of cell morphology of control (A20^{FL}) and A20$^{Cx3Cr1\text{-}KO}$ microglia from mice either or not injected with LPS. Scale bars represent 10 µm.

Supplementary Figure 5

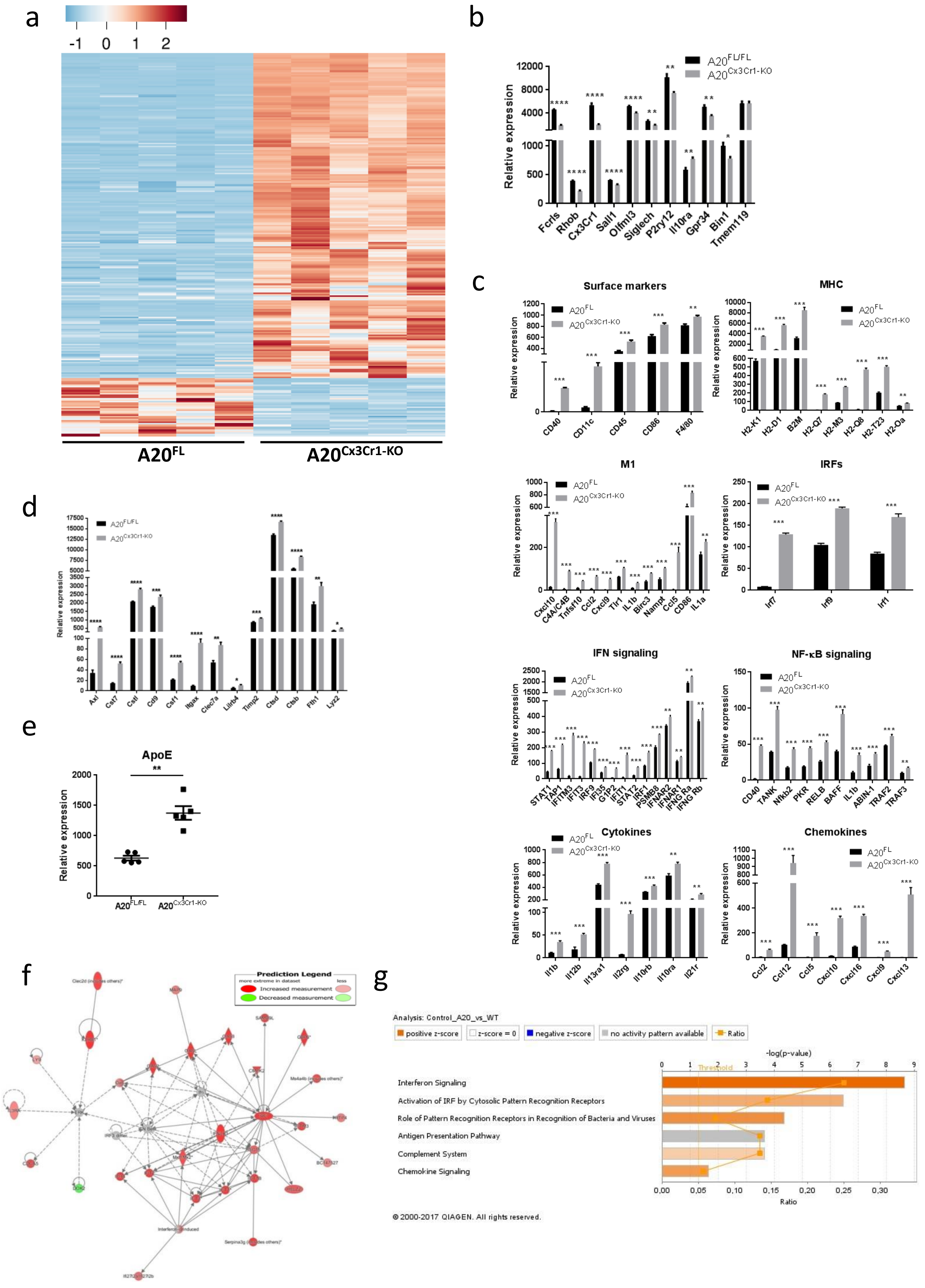

Supplementary figure 5. (**a**) Heat map of expression values for genes that are significantly (** $p < 0.01$) and at least fourfold up- or downregulated in A20$^{Cx3Cr1\text{-}KO}$ microglia compared with control (A20^{FL}) microglia. Each column represents microglia data from one individual mouse, with five mice per group. Color code presents linear scale. (**b**) mRNA expression levels of homeostatic microglial genes, and (**c**) of surface markers, microglia polarization genes, MHC class I, interferon, and inflammatory signaling genes in microglia from A20$^{Cx3Cr1\text{-}KO}$ mice compared to control mice. (**d**) mRNA expression levels of disease-associated (DAM) genes, and (**e**) *Apoe* mRNA expression in microglia from A20$^{Cx3Cr1\text{-}KO}$ mice compared to control mice. Data are expressed as mean ± SEM with four-five samples per group. Significant differences are determined by an unpaired t test (* $p < 0.05$, ** $p < 0.01$, *** $p < 0.001$, **** $p < 0.0001$). (**f**) Ingenuity Pathway Analysis identifying 'Antimicrobial response, inflammatory response, cell signaling' as the most significant network upregulated in microglia from A20$^{Cx3Cr1\text{-}KO}$ mice compared to control microglia. (**g**) Ingenuity Pathway Analysis displaying canonical pathways significantly activated in microglia from A20$^{Cx3Cr1\text{-}KO}$ mice compared to control microglia. The height of the bars indicates the significance of the overlap of molecules in the dataset to the pathways in the QIAGEN Knowledge Base. The color of the bars indicates whether the pathway is predicted to be activated (orange bars), or if the pathway is ineligible for such an assessment (gray bars), based on the z score. The orange line is the ratio, *viz.* the amount of genes in a given pathway that meet the cutoff criteria, divided by the total amount of genes that make up that pathway and that are in the reference gene set.

Supplementary Figure 6

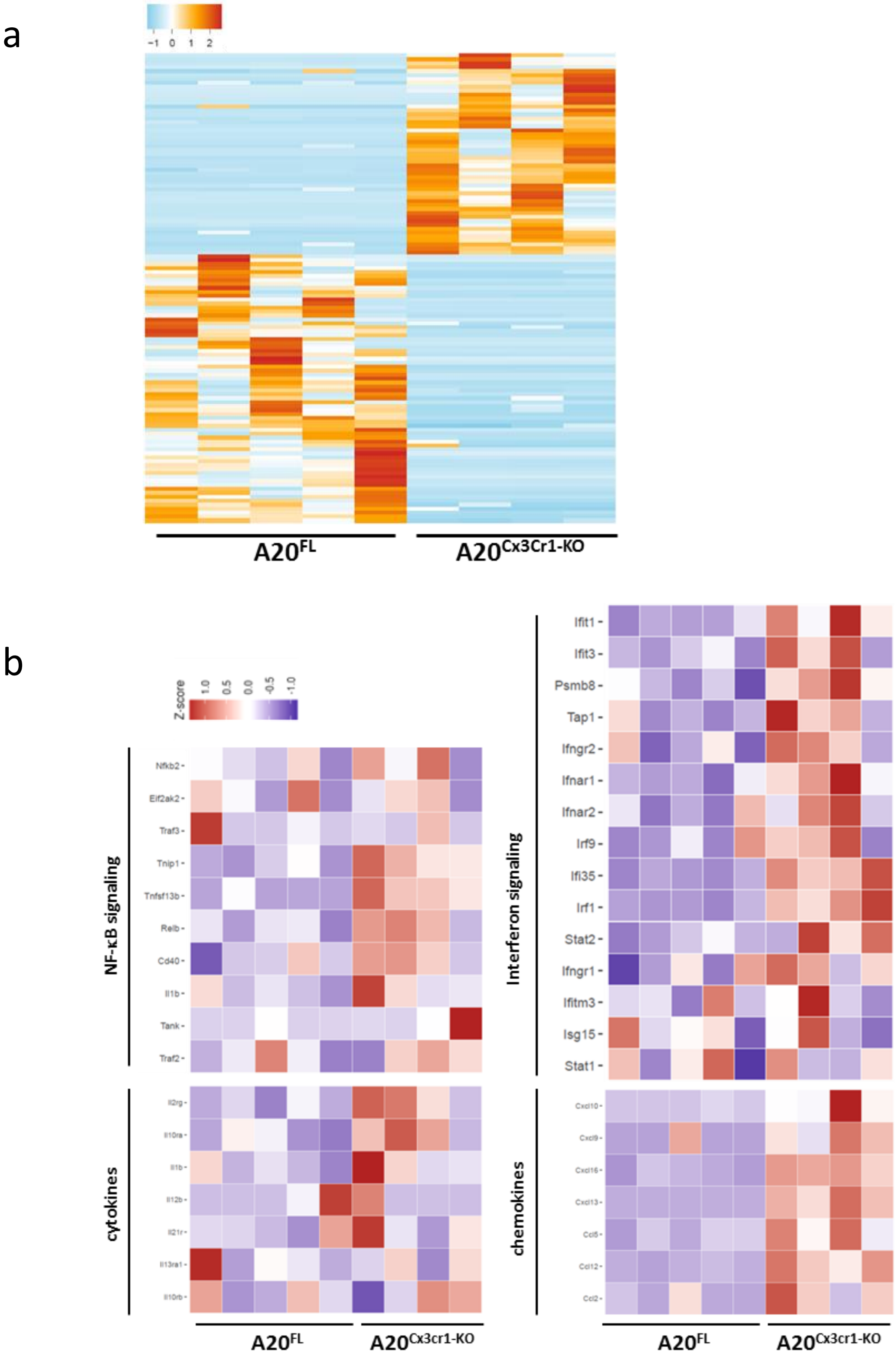

Supplementary figure 6. RNA prepared from FACS-sorted CD45^{hi}CD11b^{+}CD206^{+} macrophages from TAM-injected control (A20^{FL}) and A20$^{Cx3Cr1\text{-}KO}$ mice was submitted for RNA sequencing. (**a**) Heat map of expression values for genes that are significantly (** $p < 0.01$) and at least fourfold up- or downregulated in A20$^{Cx3Cr1\text{-}KO}$ CD45^{hi}CD11b^{+}CD206^{+} macrophages compared with control (A20^{FL}) CD45^{hi}CD11b^{+}CD206^{+} macrophages. (**b**) Pathway analysis of RNA-Seq datasets demonstrating upregulation of inflammatory pathways in CD45^{hi}CD11b^{+}CD206^{+} macrophages from A20$^{Cx3Cr1\text{-}KO}$ mice. Each column represents microglia data from one individual mouse, with four-five mice per group. Color code presents linear scale.

Supplementary Figure 7

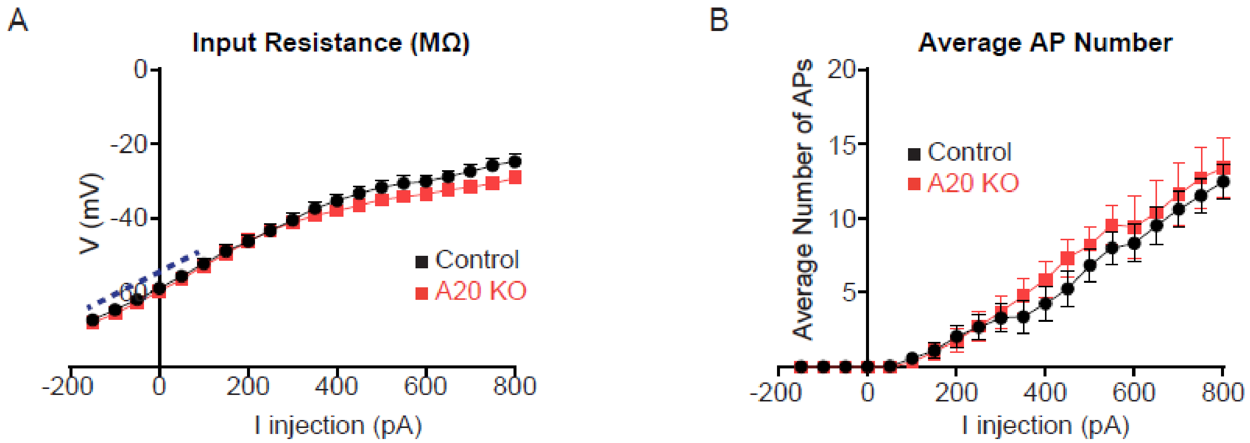

Supplementary figure 7. Microglia hyperactivation does not affect intrinsic properties of somatosensory pyramidal neurons. (**a**) Input resistance, which reflects passive membrane conductance - primarily through ion channels – is comparable between control and $A20^{Cx3Cr1\text{-}KO}$ neurons. Fitted input resistance value (dashed blue line) available in Table 1. Control, n/m=21/3; $A20^{Cx3Cr1\text{-}KO}$, n/m=20/3. Data represents means ± SEM. Number cells (n), number of animals (m) (**b**) Average number of action potentials - in response to increasing current injections – is comparable between control and $A20^{Cx3Cr1\text{-}KO}$ neurons. Control, n/m=21/3; $A20^{Cx3Cr1\text{-}KO}$, n/m=20/3. Data represents means ± SEM. Number cells (n), number of animals (m).

Supplementary Figure 8

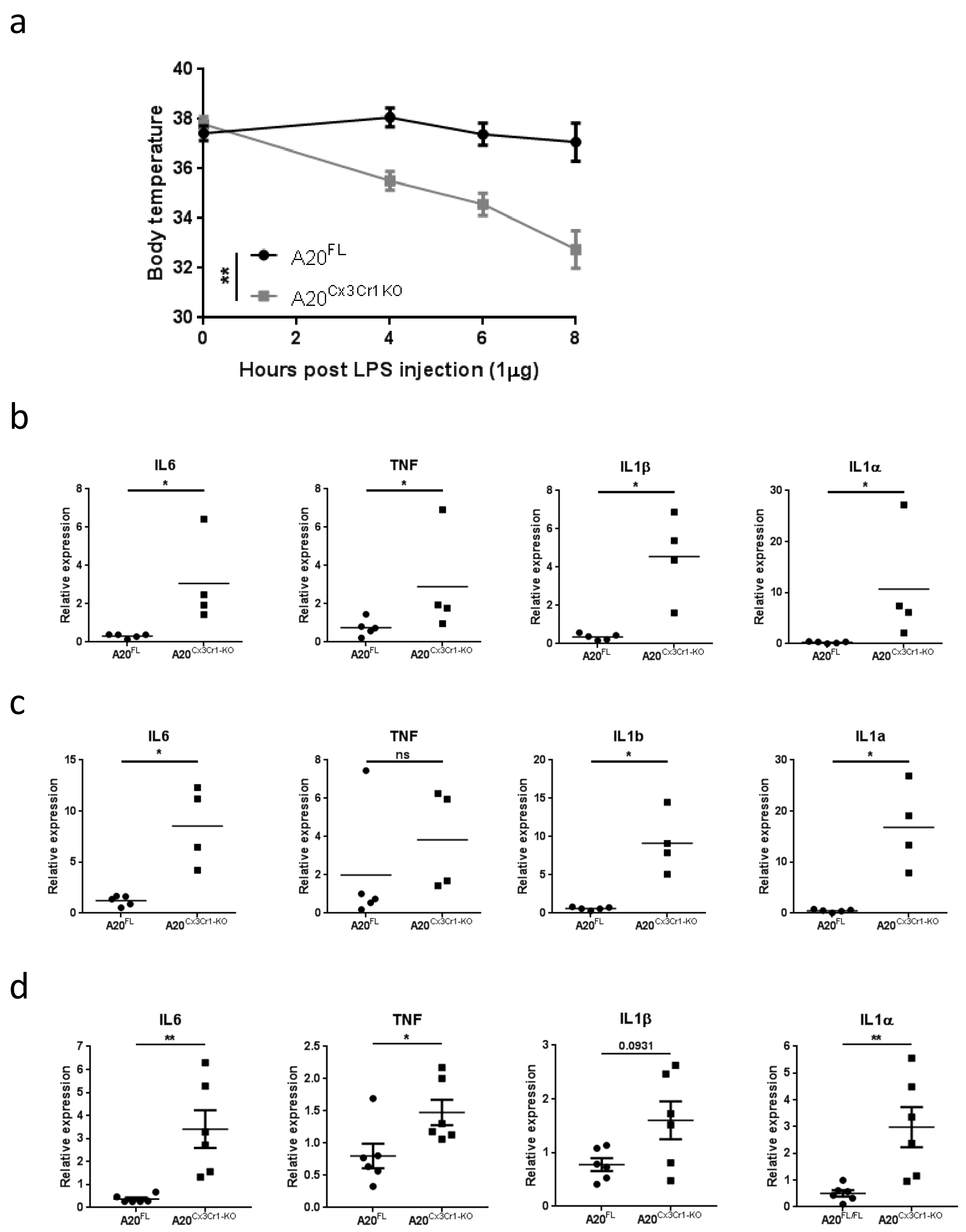

Supplementary figure 8. (a) Rectal body temperature responses were analyzed in function of time in control ($A20^{FL}$; n=7) and $A20^{Cx3Cr1\text{-}KO}$ (n=7) mice after icv injection of 1 µg of LPS. The combined results of two independent experiments are shown. Body temperature data are means ± SEM. Statistical differences were determined by a REML analysis(** $p < 0.01$). **(b-c)** Gene expression levels of inflammatory cytokines in the brain **(b)** and spinal cord **(c)** of control ($A20^{FL}$) and $A20^{Cx3Cr1\text{-}KO}$ mice 10 hours after systemic LPS injection. **(d)** Gene expression levels of inflammatory cytokines in the brain of control ($A20^{FL}$) and $A20^{Cx3Cr1\text{-}KO}$ mice 8 hours after icv injection of LPS. Each symbol represents one mouse. Data are expressed as the ratio of the mRNA expression normalized to endogenous housekeeping genes and expressed as mean ± SEM. Significant differences are determined by a Mann-Whitney U statistical test (* $p < 0.05$, ** $p < 0.01$).

Supplementary Figure 9

a

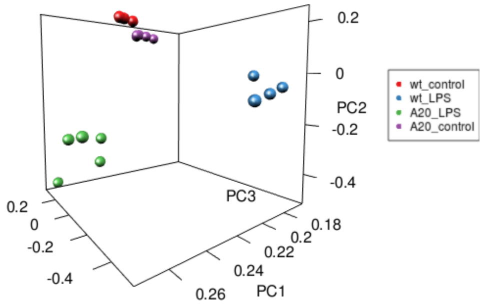

b

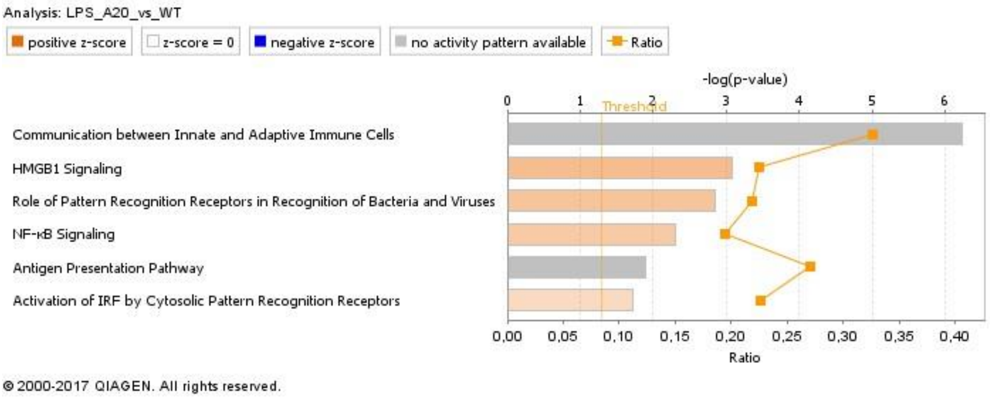

c

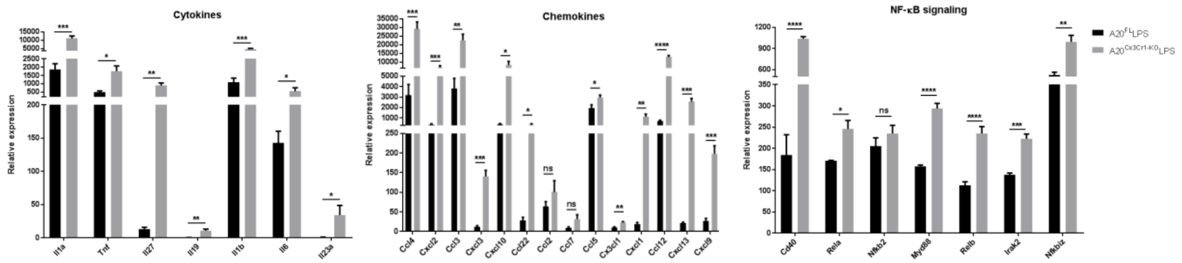

d

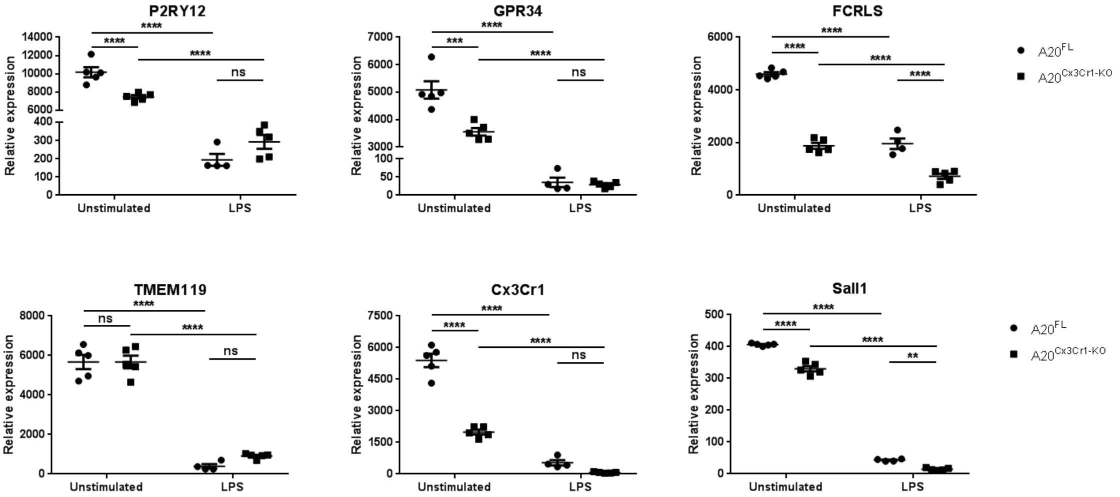

Supplementary figure 9. **(a)** Principle component analysis of transcriptional profiles of $A20^{Cx3Cr1\text{-}KO}$ microglia compared to control ($A20^{FL}$) microglia either or not stimulated with LPS for 10 h. Each point represents microglia data from one individual mouse, with four to five mice per group. (**b**) Ingenuity Pathway Analysis displaying canonical pathways significantly activated in microglia from $A20^{Cx3Cr1\text{-}KO}$ mice compared to control microglia 10 hours after LPS injection. The height of the bars indicates the significance of the overlap of molecules in the dataset to the pathways in the QIAGEN Knowledge Base. The color of the bars indicates whether the pathway is predicted to be activated (orange bars), or if the pathway is ineligible for such assessment (gray bars), based on the z score. The orange line is the ratio, *viz.* the amount of genes in a given pathway that meet the cutoff criteria, divided by the total amount of genes that make up that pathway and that are in the reference gene set. (**c**) mRNA expression levels of inflammatory signaling genes in microglia from LPS stimulated $A20^{Cx3Cr1\text{-}KO}$ mice compared to control mice. Data are expressed as mean ± SEM with four-five samples per group. Significant differences are determined by an unpaired t test (* $p < 0.05$, ** $p < 0.01$, *** $p < 0.001$, **** $p < 0.0001$). (**d**) mRNA expression levels of homeostatic microglial genes in microglia from control and $A20^{Cx3Cr1\text{-}KO}$ mice either or not stimulated with LPS. Data are expressed as mean ± SEM with four-five samples per group. Significant differences are determined by a two-way ANOVA with Sidak correction for multiple comparison (** $p < 0.01$, *** $p < 0.001$, **** $p < 0.0001$).

Supplementary Figure 10

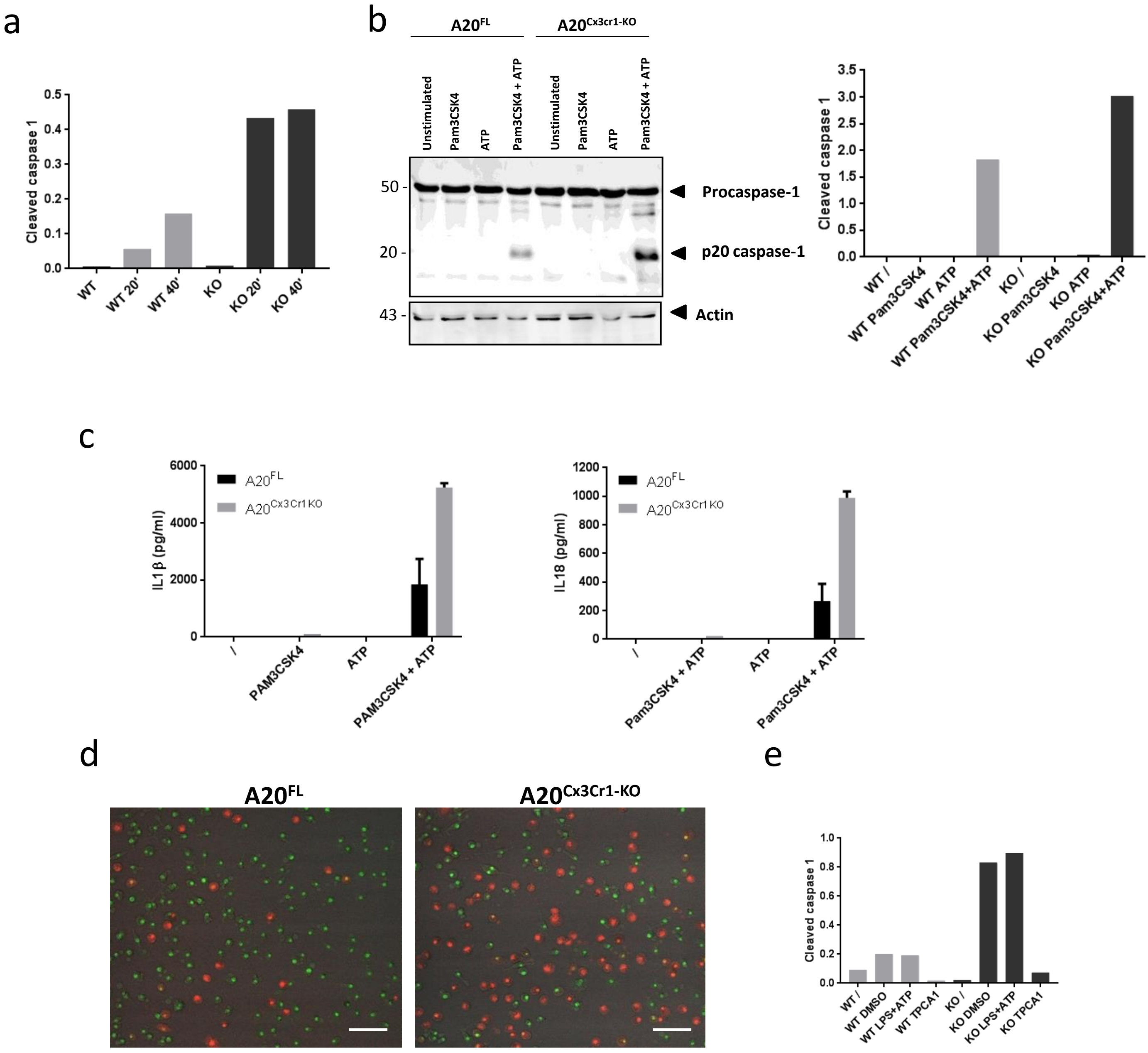

Supplementary figure 10. (**a**) Quantification of cleaved caspase-1 (p20) in primary cultured microglia from control (A20^{FL}) and A20$^{Cx3Cr1-KO}$ mice stimulated with LPS and/or ATP, as shown on immunoblot in Fig. 4c. (**b**) Immunoblot and quantification for procaspase-1 processing in primary cultured microglia from control (A20^{FL}) and A20$^{Cx3Cr1-KO}$ mice stimulated with Pam3CSK4 and/or ATP. (**c**) IL1β and IL18 protein levels in the supernatant of primary cultured microglia stimulated with Pam3CSK4 and/or ATP. Data represent the mean ± SD of two technical replicates of pooled microglial cells from control (A20^{FL}) and A20$^{Cx3Cr1-KO}$ mice. (**d)** Pyroptosis induction in primary microglia from control and A20$^{Cx3Cr1-KO}$ mice stimulated with LPS and ATP, as measured by Propidium Iodide uptake 30 min post ATP stimulation. Representative images of two independent experiments. No cell death could be detected in cells stimulated with LPS alone. Scale bar, 100 µm. (**e**) Quantification of cleaved caspase-1 (p20) in primary cultured microglia from control (A20^{FL}) and A20$^{Cx3Cr1-KO}$ mice either or not pretreated in vitro with TPCA-1 and stimulated with LPS and/or ATP, as shown on immunoblot in Fig. 4e.

Supplementary Figure 11

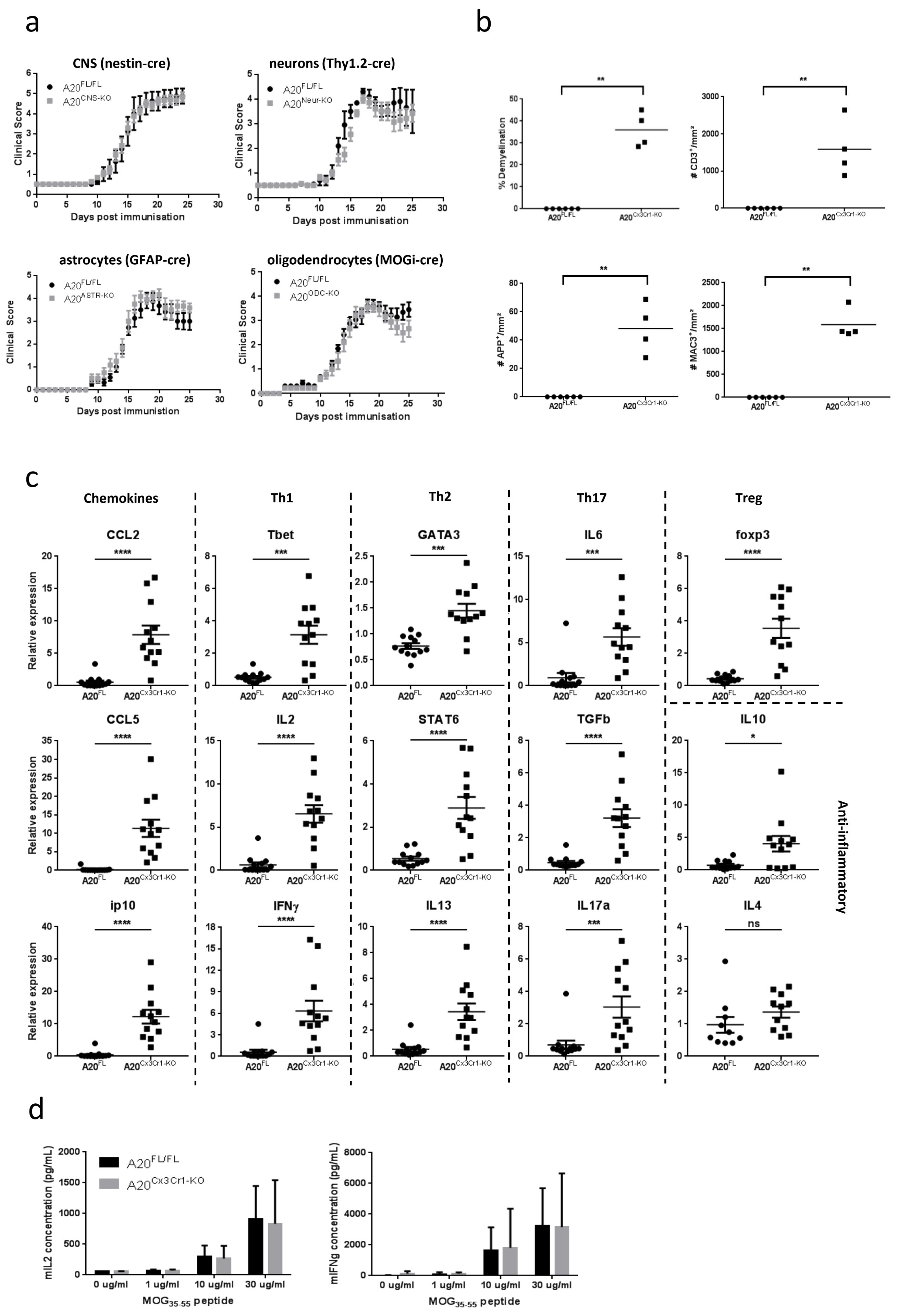

Supplementary figure 11. (a) EAE was induced by active immunization of CNS-specific A20 deficient ($A20^{CNS-KO}$, n=7), neuron-specific A20 deficient ($A20^{Neur-KO}$, n=7), astrocyte-specific A20 deficient ($A20^{ASTR-KO}$, n=6), oligodendrocyte-specific A20 deficient ($A20^{ODC-KO}$, n=9) mice and respective control littermate $A20^{FL}$ mice (n=7, 6, 11 and 13 respectively) with MOG peptide, and clinical disease development was followed over time. Each data point represents the mean ± SEM. Representative experiment from three independent experiments. **(b)** Quantification of immune cell infiltration, demyelination and neuronal damage in the spinal cord of control ($A20^{FL}$) and $A20^{Cx3Cr1-KO}$ mice 13 days post-immunization. Each symbol indicates the mean of one mouse. Significant differences are determined by a Mann-Whitney U statistical test (** $p < 0.01$). **(c)** Expression of chemokines, TH1-, TH17-, TH2-, and Treg-related factors, and anti-inflammatory cytokines in the spinal cord of control ($A20^{FL}$) and $A20^{Cx3Cr1-KO}$ mice 12 days post immunization. Each symbol represents one mouse. Data are expressed as the ratio of the mRNA expression normalized to endogenous housekeeping genes and expressed as mean ± SEM. Significant differences are determined by a Mann-Whitney U statistical test (* $p < 0.05$, *** $p < 0.001$, **** $p < 0.0001$). **(d)** Recall assay in $A20^{Cx3Cr1}$ mice. Splenocytes from MOG_{35-55} peptide-immunized $A20^{FL}$ and $A20^{Cx3Cr1KO}$ mice were cultured and stimulated with indicated MOG_{35-55} concentrations. Culture supernatants were analyzed for IL-2 and IFNγ 48h after stimulation using ELISA. Data represent mean ± SEM of 4 mice per group.

Supplementary Figure 12

a

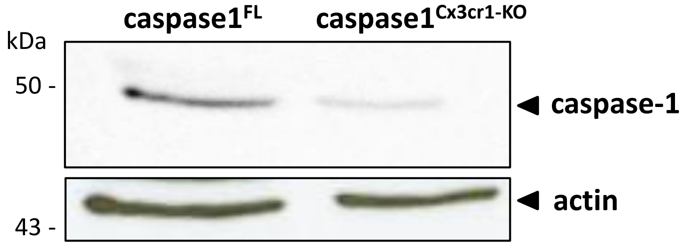

b

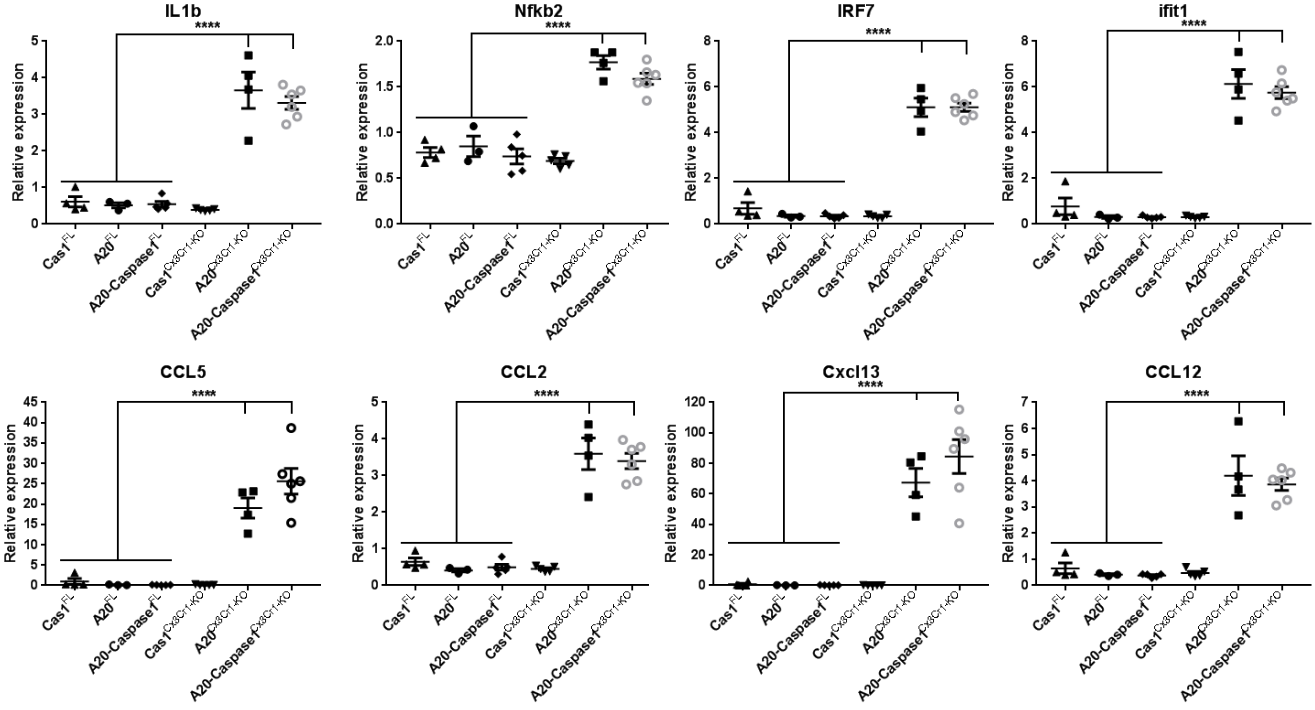

Supplementary figure 12. (**a**) Caspase-1 expression by Western blotting on *ex vivo* FACS-sorted microglia from control (caspase1^{FL}) and caspase1$^{Cx3Cr1\text{-}KO}$ mice 4 weeks after TAM injection. Actin is shown as loading control. **(b)** Expression of inflammatory genes in FACS-sorted microglia from TAM-injected control (caspase1^{FL}) and caspase1$^{Cx3Cr1\text{-}KO}$ mice. Each symbol represents one mouse. Data are expressed as the ratio of the mRNA expression normalized to endogenous housekeeping genes and expressed as mean ± SEM. Significant differences are determined by a Mann-Whitney U statistical test (**** $p < 0.0001$).

Supplementary Figure 13

Fig 1b

4w post-TAM anti-A20 | 35w post-TAM anti-Actin | 35w post-TAM anti-A20 | 35w post-TAM anti-Actin

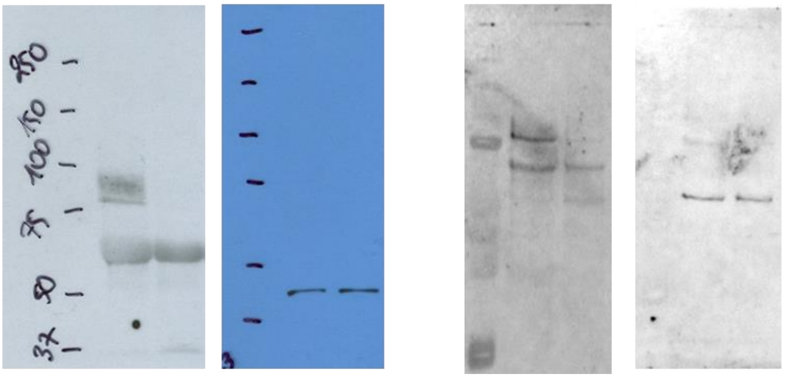

Fig 1c

A20 in vitro 4-OHTAM | Actin in vitro 4-OHTAM

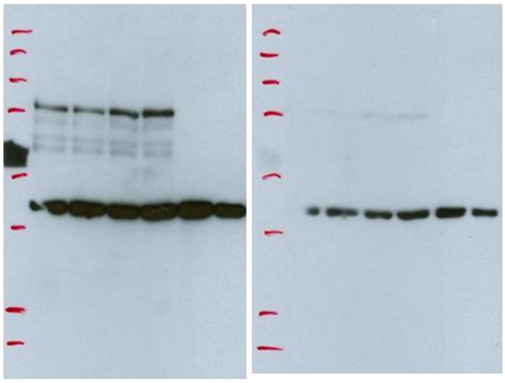

Fig 4c

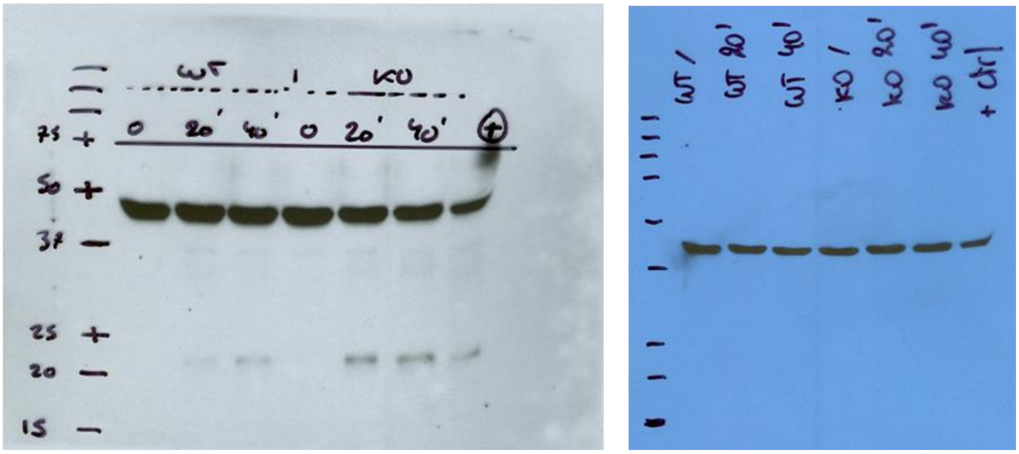

Fig 4e

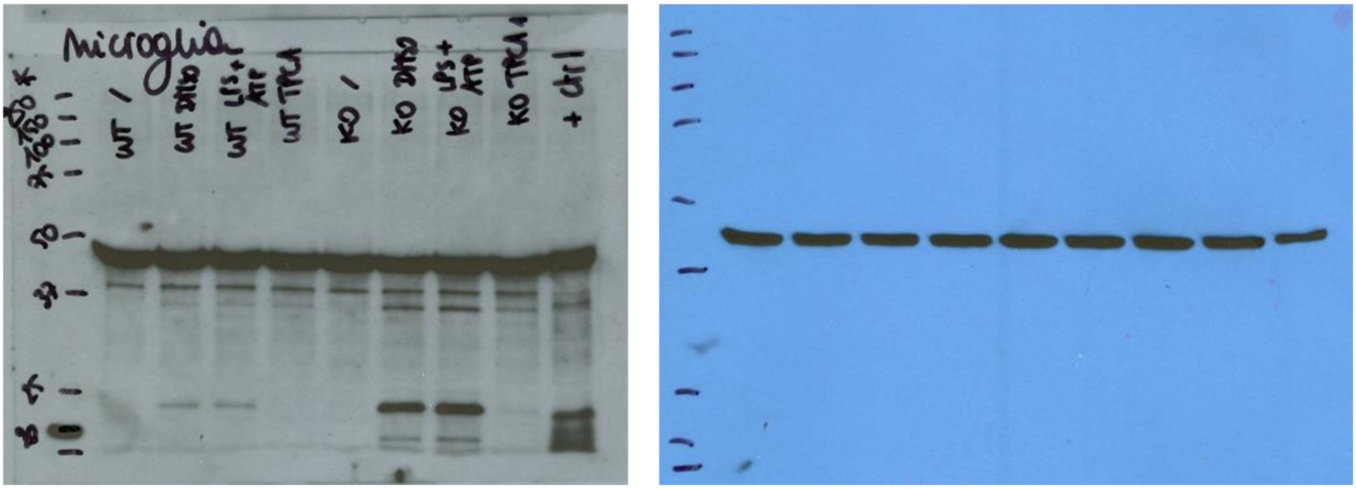

Supplementary figure 13. Full-length pictures of blots.

Supplementary Table 1. Top 6 upstream regulators using IPA core analysis to predict the likely cause of gene expression changes observed in microglia from A20$^{Cx3Cr1-KO}$ versus A20^{FL}. Expr log ratio showing the expression of the upstream regulators in the dataset. Z score showing the activation state of the upstream regulators (significantly activated if the z score is ≥ 2, significantly inhibited if the z-score ≤ -2). P value of overlap showing the overlap between the dataset genes and the genes that are regulated by a transcriptional regulator.

Upstream regulator	Expr log ratio	Z score	p value of overlap	Target molecules in datatset
Ifnar		5,979	1,57E-45	ZBP1,XAF1,VCAM1,USP18,TNFSF10, TAP1, STAT2, STAT1, Sp100, RSAD2, OASL, OAS2, OAS1, NLRC5, Mx1/Mx2, ISG20, IRF7, IFITM3, IFIT3, IFIT2, IFIT1B, IFI16, HLA-G, HLA-A, GBP2, DDX58, Cxcl9, Cxcl10, CD74, CD40, CD274, CCL5, CCL2, C3, Bst2, AXL
IFNG		8,037	1,44E-44	VCAM1, USP18, TNFSF10, THBS1, Tgtp1/Tgtp2, TAP1, STX11, STAT2, STAT1, SLFN5, Slfn1, Serpina3g, SEMA4A, RUNX3, RTP4, RSAD2, P2RY14OASL, OAS3, OAS2, Oas1b, OAS1, NLRC5, Mx1/Mx2, MAFF, Ly6a, LGALS3BP, KMO, Klra17, ITGAX, ITGAL, ISG20, ISG15, IRF7, IL3RA, IL18BP, IL12RB2, Iigp1, IFITM3, IFITM2, IFIT3, IFIT2, IFIT1, Ifi47, IFI44, IFI16, HLA-G, HLA-A, HERC6, HCAR2, Gvin1, GFAP, GCK, Gbp8, GBP6, GBP2, FPR2, FOS, FGL2, FCGR3A/FCGR3B, EPOR, EDNRB, DDX58, CYBB, Cxcl9, CXCL16, Cxcl11, CXCL10, CMPK2, CFB, CD74, CD5L, CD40, CD274, Ccl7, CCL5, CCL2, C4A/C4B, C3, C2, Bst2, BRIP1, BCL3, ASS1, ADRA2A, TMOD1, SCNN1A, Ms4a4b, MLANA, LOX, KDR, CXCR4, CD72, CCND2, ALDH1L1, ADORA1, PDCD1
IRF7	4,273 up	6,239	1,69E-43	ZBP1, XAF1, USP18, TRAF1, TNFSF10, TAP1, STAT2, STAT1, Slfn1, SAMD9L, RTP4, RSAD2, PLAC8, PHF11, Oasl2, OASL, OAS3, OAS2, OAS1, Mx1/Mx2, Ms4a4b (includes others), ITGAX, ISG20, ISG15, IRF7, IFITM3, IFITM2, IFIT3, IFIT2, IFIT1B, IFIT1, Ifi47, IFI44, IFI16, HELZ2, DHX58, DDX58, CXCL10, CMPK2, CD69, CD40, CCL5, BC147527, MICB
LPS		7,569	4,41E-41	XDH, XCR1, XAF1, VCAM1, USP18, ULBP1, TRAF1, TNFSF10, THBS1, Tgtp1/Tgtp2, TAP1, STAT2, STAT1, Slfn2, SLFN13, Slfn1, SLCO2A1, RSAD2, RAB37, PDYN, PDCD1, OGN, OASL, OAS3, OAS2, OAS1, NLRC5, Mx1/Mx2, MET, MAFF, Ly6a (includes others), LGALS3BP, KMO, KDR, ITGAX, ITGAL, ISG20, ISG15, IRF7, IL3RA, IL2RG, IL12RB2, Iigp1, IFIT3, IFIT2, IFIT1B, IFIT1, Ifi47, IFI44, HSH2D, HLA-A, HCAR2, GFAP, GBP6, GBP2, FPR2, FOS, DRAM1, DHX58, DDX58, CYP4F2, CYBB, CXCR4, Cxcl9, CXCL16, CXCL13, Cxcl11, CXCL10, CMPK2, CFB, CD74, CD69, CD40, CD300LF, CD274, CD22, CCND2, Ccl7, CCL5, Ccl2, CCL2, C3, BCL3, ASS1, ADORA1, SCNN1A, RXRG, PTGER4, NR4A2, LGALS1, KCNJ10, IFITM2, H2-T10, GAS1, CUX2, CDH5, ADRA2A, CFP, COL9A3, EHD1, H2-M2, H2-Q5, IFI16, Oas1b, SLAMF7, STX11
ACKR2	1,526 up	-5,099	3,81E-40	USP18, STAT2, STAT1, RSAD2, Oasl2, OASL, OAS3, OAS2, OAS1, ISG20, ISG15, IRF7, Iigp1, Ifitm1, IFIT3, IFIT2, IFIT1B, Ifi47, IFI44, Ifi27l2a/Ifi27l2b, IFI16, DHX58, DDX58, Cxcl9, CXCL10, CCL5
IFNB1	4,827 up	5,752	1,65E-39	ZBP1, XAF1, TNFSF10, Tgtp1/Tgtp2, STAT2, STAT1, PDCD1, Oasl2, OAS2, Oas1b, OAS1, Mx1/Mx2, MLANA, ISG20, ISG15, IRF7, Ifitm1, IFIT3, IFIT2, IFIT1B, IFIT1, IFI16, HLA-A, Gvin1 (includes others), Gbp8, GBP6, GBP2, DHX58, DDX58, CXCL10, CD40, CD274, CCL5, Ccl2, CCL2, Bst2, AXL, FOS, CMPK2, GAS1, Ifi27l2a/Ifi27l2b, Ifi47, ITGAX, Oas1d (includes others), OGN, RSAD2, SLFN13, THBS1, USP18

Supplementary Table 2. Altered gene profile in CD45^{hi}CD11b^{+}CD206^{+} macrophages compared to control CD45^{hi}CD11b^{+}CD206^{+} macrophages. Significantly up- and down- regulated genes ($p < 0.01$ and fourfold change). The significance of genes is indicated by the given p values.

GeneID	Symbol	Length	logFC	AveExpr	t	P.Value	adj.P.Val	B
ENSMUSG00000004612	Nkg7	914	10,86756	0,13989	8,675449	1,15E-07	0,000119	7,459916
ENSMUSG00000015437	Gzmb	1418	10,17253	1,476272	8,405491	1,79E-07	0,000174	7,075229
ENSMUSG00000024535	Snx24	1880	10,17058	0,775046	10,89206	4,15E-09	1,82E-05	10,14663
ENSMUSG00000042385	Gzmk	1389	9,874547	-1,29871	7,984127	3,63E-07	0,00029	6,46804
ENSMUSG00000032094	Cd3d	1323	9,683145	-1,58362	7,451056	9,21E-07	0,000645	5,617713
ENSMUSG00000053044	Cd8b1	1483	9,315384	-1,93304	8,269212	2,24E-07	0,000197	6,727553
ENSMUSG00000076498		692	9,240048	-0,77819	6,661076	3,92E-06	0,002152	4,290681
ENSMUSG00000032035	Ets1	8476	9,017318	1,66095	8,790487	9,53E-08	0,000112	7,302811
ENSMUSG00000076752		914	8,80532	-3,1096	10,41562	8,11E-09	2,6E-05	8,815322
ENSMUSG00000022584	Ly6c2	2740	8,741222	0,638923	6,929014	2,38E-06	0,001491	4,652775
ENSMUSG00000043068	Fam89a	1338	8,656533	-2,30947	6,302367	7,78E-06	0,003798	3,524524
ENSMUSG00000024910	Ctsw	1232	8,622951	-2,47824	6,759325	3,26E-06	0,001908	4,271115
ENSMUSG00000000409	Lck	2929	8,58539	0,965858	6,122216	1,11E-05	0,005249	3,279068
ENSMUSG00000022586	Ly6i	1156	8,448262	3,28168	9,922842	1,66E-08	3,64E-05	8,307101
ENSMUSG00000032093	Cd3e	1436	7,94773	-0,75451	5,745471	2,34E-05	0,009119	2,611968
ENSMUSG00000090231	Cfb	2825	7,917449	2,946471	9,623278	2,6E-08	4,56E-05	7,791831
ENSMUSG00000059412	Fxyd2	632	7,733129	1,203337	6,312856	7,62E-06	0,003798	3,508915
ENSMUSG00000054931	Zkscan4	2400	7,636508	-2,8562	5,922906	1,64E-05	0,007021	2,754302
ENSMUSG00000104713	Gbp6	5954	7,04086	1,718199	8,350763	1,96E-07	0,000181	6,000075
ENSMUSG00000024143	Rhoq	4151	6,796781	3,585855	5,765378	2,24E-05	0,008961	2,498603
GeneID	Symbol	Length	logFC	AveExpr	t	P.Value	adj.P.Val	B
ENSMUSG00000021675	F2rl2	2502	-10,1009	-0,56144	-11,9595	1E-09	5,87E-06	10,95876
ENSMUSG00000056220	Pla2g4a	6391	-9,99178	2,066294	-9,02931	6,51E-08	8,17E-05	7,785226
ENSMUSG00000049225	Pdp1	4507	-9,91204	0,388394	-9,16095	5,29E-08	7,15E-05	7,912859
ENSMUSG00000065987	Cd209b	2115	-9,84039	1,17188	-5,92972	1,62E-05	0,007021	3,09027
ENSMUSG00000040703	Cyp2s1	4938	-9,61806	-1,8467	-14,6902	4,02E-11	7,07E-07	12,72932
ENSMUSG00000031495	Cd209d	919	-9,58249	2,342083	-10,3321	9,14E-09	2,6E-05	9,281455
ENSMUSG00000035790	Cep19	1751	-9,28693	0,881843	-9,21248	4,88E-08	7,15E-05	7,883621
ENSMUSG00000030201	Lrp6	13672	-9,27738	1,994656	-9,68213	2,38E-08	4,56E-05	8,417152
ENSMUSG00000021578	Ccdc127	9559	-9,17047	3,077419	-10,2464	1,03E-08	2,6E-05	8,963459
ENSMUSG00000031600	Vps37a	6379	-8,91752	2,695709	-6,68487	3,75E-06	0,002124	4,287287
ENSMUSG00000038914	Dido1	13307	-8,91451	1,31378	-7,68682	6,07E-07	0,000464	5,755638
ENSMUSG00000037722	Gnpnat1	2629	-8,89236	0,836547	-9,42618	3,51E-08	5,6E-05	7,944519
ENSMUSG00000013833	Med16	4663	-8,67819	2,856762	-5,6983	2,57E-05	0,00981	2,61419
ENSMUSG00000070524	Fcrlb	1284	-8,59853	2,729866	-13,1454	2,32E-10	2,04E-06	11,11519
ENSMUSG00000005417	Mprip	13327	-8,26316	2,896624	-6,34527	7,16E-06	0,003701	3,617125
ENSMUSG00000036435	Exoc1	12042	-8,25057	1,671334	-7,4308	9,54E-07	0,000645	5,197234
ENSMUSG00000079481	Nhsl2	16952	-8,24943	-1,5076	-6,82448	2,88E-06	0,001748	4,32803
ENSMUSG00000066571	4931406P16Rik	8239	-8,23166	0,563097	-8,10295	2,97E-07	0,000248	5,973407
ENSMUSG00000027715	Ccna2	4083	-8,17979	0,570871	-5,97758	1,47E-05	0,006622	2,976163
ENSMUSG00000026073	Il1r2	1908	-8,08907	4,664322	-7,66146	6,35E-07	0,000465	5,363868
ENSMUSG00000001552	Jup	6436	-7,9456	2,650556	-6,97176	2,19E-06	0,001428	4,372837
ENSMUSG00000059474	Mbtd1	13896	-7,79447	2,538718	-8,69116	1,12E-07	0,000119	6,678842
ENSMUSG00000020220	Vps13d	24771	-7,12361	1,459847	-5,86397	1,84E-05	0,007707	2,674644
ENSMUSG00000029119	Man2b2	3528	-5,96267	1,504724	-6,05198	1,27E-05	0,005868	2,735103

Supplementary Table 3. Detailed information on patient data (age, gender, diagnosis, clinical disease symptoms) samples (post-mortem tissue and CSF samples).

Frozen Tissue					
**Patient ID**	**Age (years)**	**Gender**	**Diagnosis**	**MS detail**	**Clinical**
4887	28	F	Multiple Sclerosis		Depression, Pneumonia, Tobacco abuse, Osteoporosis, Dysphagia, Paraperesis, Chronic urinary tract infection
5352	49	F	Multiple Sclerosis		Lyme Disease, possible head trauma, History of chronic urinary tract infection
5170	52	F	Multiple Sclerosis	(chronic progressive)	Optic neuritis, Depression
4864	53	M	Multiple Sclerosis		Diabetes Type I, Seizure disorder, Depression, Cholecystiits, Hypothyroidism, Leukocytosis, Pneumonia, Sepsis, Chronic urinary tract infection
5252	49	M	Multiple Sclerosis	(2° Progressive)	
5276	42	F	Multiple Sclerosis	(2° Progressive)	
5352	49	F	Multiple Sclerosis		Lyme Disease, possible head trauma, History of chronic urinary tract infection
4494	67	M	Normal		CA, prostate, Depression, Dementia, History of Aneurysm, Hypertension, Chronic urinary tract infection
4956	92	F	Normal		CA uterine, CA stomach, Congestive heart failure, Hypertension, Macular degeneration, Asthma, Osteoporosis
5214	61	M	Normal		Normal
5072	83	M	Normal		Chronic obstructive pulmonary disease, Seizure disorder (clinical only), Atrial fibrillation

CSF					
**Patient ID**	**Age (years)**	**Gender**	**Diagnosis**	**MS detail**	**Clinical**
13580	53	M	Multiple Sclerosis		Diabetes mellitus, Seizure, Hypothyroidism, Toxoplasmosis
13591	28	F	Multiple Sclerosis		
13647	82	M	Multiple Sclerosis	(chronic progressive)	
13649	52	F	Multiple Sclerosis	(chronic progressive)	
13657	49	F	Multiple Sclerosis	(chronic progressive)	
13601	63	F	Multiple Sclerosis		Depression, Optic neuritis, Spondylosis, cervical, Seizure, Pneumonia, Neuralgia, trigeminal, Ataxia, Infection, urinary tract, Neurogenic bladder
13543	58	F	Multiple Sclerosis	Chronic MS plaque formation	Pneumonia, Infection, urinary tract
13552	38	F	Multiple Sclerosis	Chronic-active MS plaque formation	Encephalitis, Herpes zoster, Pneumonia
13629	65	M	Multiple Sclerosis	(2° progressive) Chronic MS plaque formation	Quadriplegia, Depression, Optic neuritis, COPD
13354	76	M	Normal		Dementia, Diabetes mellitus, Renal Failure
13376	49	M	Normal		CA, colon
13264	67	M	Normal		Dementia, CA, prostate, Depression, Aneurysm, Hypertension
13203	68	F	Normal		Heart attack
