# Supplementary material for: A20 critically controls microglia activation and inhibits inflammasome-dependent neuroinflammation
Source: Nat Commun. 2018 May 23;9:2036. doi: 10.1038/s41467-018-04376-5 (PMC5964249; doi:10.1038/s41467-018-04376-5)
Supplement: Supplementary file 3 — Description of Additional Supplementary Files [file 41467_2018_4376_MOESM3_ESM.pdf]

Description of Additional Supplementary Files

File Name: Supplementary Data 1
Description: Altered microglial gene profile in A20Cx3Cr1-KO microglia compared to control microglia. Significantly up- and down- regulated genes ($p < 0.01$ and fourfold change). The significance of genes is indicated by the given p values.
